# Spanish Adaptation of the Organizational Culture Assessment Instrument: Reflection on the Difficulty in Transferring the *ad hoc* Factor

**DOI:** 10.3389/fpsyg.2021.817232

**Published:** 2021-12-24

**Authors:** Jordi Assens-Serra, Maria Boada-Cuerva, María-José Serrano-Fernández, Esther Villajos, Joan Boada-Grau

**Affiliations:** ^1^Department of Strategy, Leadership and People, EADA Business School, Barcelona, Spain; ^2^Human Factor, Organizations and Markets (FHOM), Faculty of Business and Economics, Universitat Rovira i Virgili (URV), Tarragona, Spain; ^3^Faculty of Psychology, Universitat Rovira i Virgili (URV), Tarragona, Spain; ^4^Department of Economics and Social Sciences, Universitat Politècnica de València, Valencia, Spain

**Keywords:** organizational culture, clan culture, *ad hoc* culture, market culture, hierarchy culture, questionnaire translation, psychometric adaptation, Spanish language

## Abstract

The Organizational Culture Assessment Instrument is a common instrument for measuring organizational culture in English-speaking countries based on four factors: Clan, *ad hoc*, Market and Hierarchy. However, to date, there is no proper translation of the scale into Spanish. In this study, we describe the translation and adaptation of the instrument through Exploratory Factor Analysis with a Spanish sample (*n*_1_ = 246; 69.9% men and 30.1% women) and Confirmatory Factor Analysis with a Peruvian sample (*n*_2_ = 510; 70.4% men and 29.6% women). The result reduces the four-factor internal structure to a three-factor structure that retains the Clan, Market and Hierarchy factors, but completely excludes the *ad hoc* factor. Confirmatory Factor Analysis shows acceptable indicators, reliabilities are good and indication of validity is also confirmed. In conclusion, this study has given rise to the instrument in Spanish, called OCAI-12, which is suitable for evaluating organizational culture.

## Introduction

Organizational culture is an important concept in research, mainly due to its impact on corporate functioning and its business results. According to Schein (1992), organizational culture is a pattern of basic presuppositions that are shared by a group and learned as they solve problems of internal integration and external adaptation. These presuppositions have worked well enough to be considered valid and are taught to new members of the organization as the correct way to perceive, think and feel in relation to these problems. The connection between individual-level actions with the collective organizational-level practices is due to owners and managers, who play a crucial role through their leadership role by engaging in activities jointly with employees and other stakeholders (Del Giudice et al., 2017).

Given the importance of organizational culture, measuring it has to enable research in promising fields where the type of culture has an important influence. For example, this happens in resource allocation decision-making processes, that have impact not only in the company itself, but also on the territory and society (Quattrociocchi et al., 2017). Organizational culture is also crucial in the development of organizational ambidexterity, where “the overall organizational culture is supportive of employees looking out for both present and future business opportunities” (Kaur et al., 2019, p. 45). Another example in which organizational culture is also playing a role is in the knowledge acquisition process and the HR practices, having an impact on innovation performance (Papa et al., 2018). A common instrument for measuring organizational culture in English-speaking countries is the Organizational Culture Assessment Instrument (OCAI, Cameron and Quinn, 2006). The OCAI is based on a theoretical model known as the Competing Values Framework (CVF, Quinn and Rohrbaugh, 1983), and can be used to measure, in a simple way, four cultural types, namely Clan, *ad hoc*, Market and Hierarchy. In many studies the OCAI is preferred to other measures of organizational culture because it (a) makes it possible to graphically represent four culture archetypes, simplifying the enormous number of dimensions of culture; (b) covers four key presuppositions that boost organizational effectiveness: collaborate, create, compete and control; (c) is parsimonious and can be easily incorporated into studies with other sets of instruments; (d) is user-friendly and easy to understand, which reduces respondents’ resistance; and (e) uses a Likert-type response format that enables individuals to obtain stable estimates of the organizational culture. The OCAI is widely used for research in English-speaking countries but has not yet been properly translated and adapted into Spanish using Confirmatory Factor Analysis. Therefore, a reliable and valid translation of the OCAI is needed to conduct research on organizational culture in Spain and Latin-America, using the Cameron and Quinn model. This also opens up the opportunity to conduct comparative studies between different countries.

According with this research problem, the first objective of this research is to analyze the internal structure of the Spanish translation of the original OCAI scale (Cameron and Quinn, 2006) through Exploratory Factor Analysis (EFA) and Confirmatory Factor Analysis (CFA). If the Spanish questionnaire has a good construct equivalence, it will have the same four factors with six items each. Our second objective is to calculate the reliability of the instrument. If the reliability of each factor is correct, the Cronbach’s alpha will be 7 or higher. And finally, our third objective is to evaluate evidence of validity using one business performance scale, two business strategy scales, two market orientation scales and two external correlates that measure the size of the company. According to the literature we expect to find these correlations:

(a) The Hierarchy culture will have a smaller correlation with business performance than the Clan and Market cultures (Deshpandé et al., 1993; Cameron and Quinn, 2006; Jogaratnam, 2017).
(b) The Hierarchy culture will have a positive correlation with the Low-cost strategy (Slater et al., 2010; Nase and Arkesteijn, 2018).
(c) The Market culture will have a positive correlation with the Prospector strategy (Cameron and Freeman, 1991).
(d) The Hierarchy culture will have a smaller correlation with Market orientation than the Clan and Market cultures (Cameron and Quinn, 2006; Iglesias et al., 2011; Gao, 2017; Jogaratnam, 2017).
(e) The Clan culture will have a negative correlation with the size of the company (Cameron and Quinn, 2006).

## Materials and Methods

The objective of this study is to translate and adapt the OCAI into Spanish. To do this, we conducted expert translation, backtranslation and discussion of the measures, followed by a pilot test. Then we adopted a quantitative approach, collecting and analyzing the necessary data.

### Participants

A total of 756 managers from diverse Spanish and Peruvian organizations participated in the research. The study differentiated between a Spanish sample (*n*_1_ = 246; 69.9% men and 30.1% women; average age 42.2 years) and a Peruvian sample (*n*_2_ = 510; 70.4% men and 29.6% women; average age 35.3 years). See Table 1 for the variables of the two samples.

**TABLE 1:** Summary of the sociodemographic variables of participants in the two samples.

Variable		Spain *n*_1_ = 246	Latin-America *n*_2_ = 510
Gender (%)	Male	69.9	70.4
	Female	30.1	29.6
Age (years)	*M*	42.2	35.3
	SD	8.1	5.8
Professional experience (years)	*M* SD	16.4 9.0	11.0 5.8
Educational level (%)	High School studies	4.3	2.2
	University studies	41.8	40.5
	Master’s degree	49.3	56.2
	Ph.D.	4.6	1.1
Job title	Managers	65.5	46.5
	Higher technicians	13.0	32.4
	Middle-level technicians	17.9	4.7
	Administrative staff	2.8	13.1
	Others	0.8	3.3
Nationalities (%)	Spain	98.4	0
	Peru	0	86.7
	Ecuador	0	6.3
	Other countries	1.6	7.0
Company type (%)	Government	5.7	9.9
	Private companies	84.5	87.7
	Foundations	9.0	2.2
	Others	0.8	0.2
Company scope (%)	Local	15.9	19.2
	Regional	10.2	9.0
	National	25.6	36.3
	Multinational	34.1	24.9
	Global	14.2	10.6
Company sector (%)	Primary	6.5	14.9
	Secondary	43.2	29,.1
	Tertiary	50.3	56,.0
Number of employees	1 to –10	15.0	10.6
	11 to –50	19.1	13.1
	51 to –500	29.7	26.9
	501 to –5,000	19.5	30.4
	More than 5,000	16.7	19.0
Company turnover	0 –100 k €	13.4	11.4
	100 k -1 M €	10.6	11.4
	1 M € - 10 M €	16.3	16.1
	10 M € - 100 M €	28.0	22.7
	100 M € -1,000 M €	12.2	17.8
	+1,000 M €	19.5	20.6

### Measures

The English version of the OCAI scale (Cameron and Quinn, 2006) has four factors each with six items: The Clan factor measures the assumption that the company will succeed based on its human capital (α = 0.74; e.g., “The management style in the organization is characterized by teamwork, consensus, and participation”); The *ad hoc* factor measures the assumption that the company will be successful thanks to its creative and innovative capacity (α = 0.79; e.g., “2. The organization is a very dynamic and entrepreneurial place. People are willing to stick their necks out and take risks”); The Market factor measures the assumption that it is necessary to compete aggressively to get business results (α = 0.71; e.g., “3. The organization is very results-oriented. A major concern is with getting the job done. People are very competitive and achievement-oriented”); and finally, the Hierarchy factor measures the assumption that success comes with stable, predictable and efficient formal rules and policies (α = 0.73; e.g., “12. The management style in the organization is characterized by security of employment, conformity, predictability, and stability in relationships”).

Evidence of validity was assessed using one scale for measuring business performance, two scales for measuring organizational strategy, two scales for measuring market orientation and two correlates. Business performance was measured with the Performance measure (Babakus et al., 1996), which measures sales volume, market share, profitability, and customer satisfaction in relation to the organization’s objectives and also the major competitor (α = 0.88; sample item: Sales volume compared to sales unit objectives). The measure uses a five-point Likert scale from 1- much worse to 5- much better.

Organizational strategy was measured using two scales. The Prospector strategy (Slater and Olson, 2000) measures the behavior of being the first to market a new product or service concept. The Low-cost strategy (Slater and Olson, 2000) focuses on producing goods or services as efficiently as possible and at the best price. Both scales use one item to measure each strategy. Market orientation was measured using two scales. Responsive Market Orientation (MORTN; Deshpandé and Farley, 1998) measures the company’s activities for discovering and satisfying the clients’ expressed needs (α = 0.88; sample item: Our business objectives are driven primarily by customer satisfaction). Proactive Market Orientation (MOPRO; Narver et al., 2004) measures the company’s activities for discovering and satisfying the hidden and unconscious needs of the clients (α = 0.86; sample item: We continuously try to discover additional needs of our customers of which they are unaware). The response format for market orientation is a five-point Likert scale (1—strongly disagree to 5- strongly agree). Finally, the correlates Annual turnover and Number of employees were used to measure the size of the company.

### Procedure

Participants were obtained through non-probabilistic sampling (Hernández et al., 2000), also called random-accidental sampling (Kerlinger, 2001). The data were collected between December 2016 and September 2017 through an online questionnaire. Participants answered voluntarily and did not receive any monetary or in-kind gratification. The response rate was 86% and there were no cases with missing data. The instruments were translated into Spanish following the steps outlined in the scientific literature (Brislin, 1970; Hambleton, 1994; Muñiz and Hambleton, 2000; Hambleton et al., 2005; Muñiz and Bartram, 2007): translation of the items into Spanish by experts and a focus group to discuss the translation, back-translation into English and verification of the equivalence between the two versions. Before administering the instruments, we conducted a pilot test with Spanish and Peruvian managers to verify that the instruments were clearly understood.

### Data Analysis

The OCAI scale was adapted to Spanish by applying EFA with the Spanish sample (*n*_1_ = 246) using the main axes extraction method and applying Promin rotation (Lorenzo-Seva, 1999), using the FACTOR 7.2 program (Lorenzo-Seva and Ferrando, 2006). Polychoric correlation matrices were used as they are especially indicated in cases where the items have a Likert response format (Muthen and Kaplan, 1992). Parallel analysis (Timmerman and Lorenzo-Seva, 2011) and the “minimum average partial” criterion of Velicer (1976) were used to assess the number of retained factors.

Confirmatory factor analysis was then conducted with the Peruvian sample (*n*_2_ = 510) using the AMOS 21.0 program. This enabled us to specify, estimate, evaluate and present a CFA model in an intuitive diagram that shows possible relationships between the variables. Structural equation modeling (SEM) has advantages for testing the properties of a scale and therefore provides a method for examining the underlying structure of latent variables. The global adjustment indexes used in the structural equation models are the root mean square error of approximation (RMSEA), the comparative fit index (CFI) and the Tucker–Lewis index (TLI). We used the SPSS program (23.0) to calculate the reliability of the factors (Cronbach’s alpha). The SPSS program was also used to explore evidence of validity, assessing the relationship of the three cultures with other scales and correlates.

## Results

### Exploratory Factor Analysis

The results of Bartlett’s sphericity test (χ2 = 3,139.7, df = 276; *p* = 0.00), the Kaiser–Meyer–Olkin (KMO) sample adequacy index of 0.869 and the coefficient of determinant (0.00000168) showed that the data were suitable for applying factor analysis. The Promin rotation method was used to establish the EFA (Lorenzo-Seva, 1999). Table 2 shows the saturation in the rotated matrix of the translated questionnaire. Parallel analysis (Timmerman and Lorenzo-Seva, 2011) and the Minimum average partial criterion of Velicer (1976) confirmed that an internal structure of three factors was adequate (Table 2). The EFA discards three items due to complexity (loadings above 0.30 in more than one factor). The total explained variance was 61.02 %.

**TABLE 2:** Saturation in the rotated matrix of the translated original 24 item OCAI, with EFA (*n*_1_ = 246) and number of the OCAI-12 items, with CFA (*n*_2_ = 510).

Clan Items (Original OCAI)	OCAI-12 item number	Factor loadings		
		(Market)	(Hierarchy)	(Clan)
1.	(e)	−**0.37**	0.01	**0.62**
2.	(c)	0.07	0.02	**0.71**
3.	Clan-1	−0.15	0.07	**0.83**
4.	Clan-2	−0.27	0.05	**0.73**
5.	Clan-3	−0.19	−0.03	**0.88**
6.	Clan-4	−0.24	0.20	**0.74**
**Ad-hoc items (Original OCAI)**				
1.	(c)	0.16	−0.15	**0.73**
2.	(c)	0.21	−0.14	**0.77**
3.	(c)	0.21	−0.08	**0.64**
4.	(c)	0.20	0.05	**0.70**
5.	(c)	0.14	−0.00	**0.73**
6.	(e)	**0.33**	−0.10	**0.40**
**Market items (Original OCAI)**				
1.	Market-1	**0.66**	0.03	0.07
2.	(c)	**0.82**	−0.05	−0.19
3.	Market-2	**0.80**	0.09	−0.06
4.	Market-3	**0.60**	0.17	0.23
5.	Market-4	**0.80**	0.05	0.01
6.	(c)	**0.70**	−0.05	−0.04
**Hierarchy items (Original OCAI)**				
1.	(c)	0.25	**0.60**	−0.18
2.	(e)	0.13	**0.51**	**0.42**
3.	Hierarchy-1	−0.30	**0.58**	0.08
4.	Hierarchy-2	0.15	**0.77**	−0.17
5.	Hierarchy-3	−0.10	**0.89**	−0.03
6.	Hierarchy-4	0.15	**0.50**	0.00

*(e) Discarded by the EFA due to complexity, (c) Discarded by the CFA. Absolute values above 0.3 are highlighted in bold.*

### Confirmatory Factor Analysis

**FIGURE 1:**
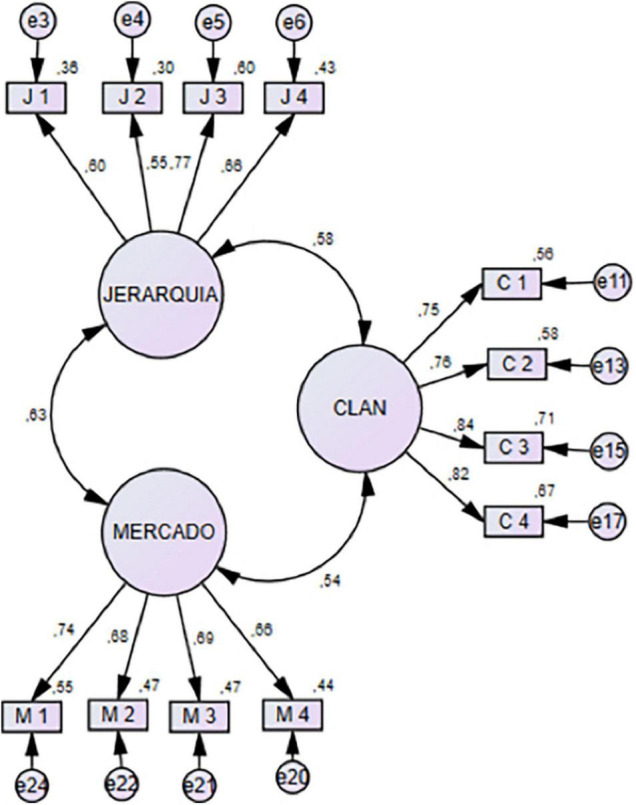
Confirmatory factor analysis of the OCAI-12 scale (*n*_2_ = 510) (Chi-square = 55.224; df = 20; *P*-value = 0.0000; TLI = 0.93; CFI = 0.94; RMSEA = 0.07).

The CFA makes it possible to identify and discard the items that prevent obtaining adequate indicators of goodness of fit. It confirmed a good adjustment of a three-factor model in which the Clan, Market and Hierarchy factors were maintained (going from six to four items each) but the *ad hoc* factor was totally excluded. The following indicators of goodness of fit were used: TLI (Tucker–Lewis Index), CFI (Comparative Fit Index) and RMSEA (Root Mean Square Error of Approximation). For the cut-off points in the adjustment indices of the structural models, there is some unanimity in the fact that values equal to or higher than 0.90 in the TLI and CFI are acceptable and are considered excellent when they exceed the value of 0.95 (Lévy-Mangin and Varela-Mallou, 2006). RMSEA is considered acceptable when it is less than 0.08 and excellent when it is equal to or less than 0.05 (Bentler, 1990; Hu and Bentler, 1999; Fan and Sivo, 2007). The indicators were close to values that are considered adequate (TLI = 0.93, CFI = 0.94, RMSEA = 0.07). Figure 1 shows the diagram of the OCAI-12 scale (*n*_2_ = 510).

Table 3 shows the 24 original OCAI items and the OCAI-12 items.

**TABLE 3:** Original OCAI items and number of the OCAI-12 items, with CFA (*n*_2_ = 510).

Clan Items (Original OCAI)	OCAI-12 item number
1. La organización es un lugar muy personal. Es como una familia ampliada. La gente comparte muchas cosas de sí mismas. (The organization is a very personal place. It is like an extended family. People seem to share a lot of themselves).	(e)
2. En general se considera que el liderazgo de la organización constituye un ejemplo de *mentoring*, facilitación y desarrollo. (The leadership in the organization is generally considered to exemplify mentoring. facilitating or nurturing).	(c)
3. El estilo directivo de la organización se caracteriza por el trabajo en equipo, el consenso y la participación. (The management style in the organization is characterized by teamwork, consensus, and participation).	Clan-1
4. Lo que mantiene la organización unida es la lealtad y la confianza mutua. El compromiso con la organización es muy alto. (The glue that holds the organization together is loyalty and mutual trust. Commitment to this organization runs high).	Clan-2
5. La organización pone énfasis en el desarrollo humano. Se mantienen unos altos niveles de confianza, apertura y participación. (The organization emphasizes human development. High trust, openness, and participation persist).	Clan-3
6. La organización define el éxito en base al desarrollo de los recursos humanos, el trabajo en equipo, el compromiso de los empleados y el interés por las personas. (The organization defines success on the basis of the development of human resources, teamwork, employee commitment, and concern for people).	Clan-4
***Ad hoc* items (Original OCAI)**	
1. La organización es un lugar muy dinámico y emprendedor. La gente está dispuesta a asumir riesgos. (The organization is a very dynamic and entrepreneurial place. People are willing to stick their necks out and take risks).	(c)
2. En general se considera que el liderazgo de la organización constituye un ejemplo de emprendeduría, innovación o asunción de riesgos. (The leadership in the organization is generally considered to exemplify entrepreneurship, innovation or risk taking).	(c)
3. El estilo directivo de la organización se caracteriza por la toma de riesgos a nivel individual, la innovación y la libertad. (The management style in the organization is characterized by individual risk taking, innovation, freedom, and uniqueness).	(c)
4. Lo que mantiene la organización unida es el compromiso con la innovación y el desarrollo. Se pone especial énfasis en estar siempre a la última. (The glue that holds the organization together is commitment to innovation and development. There is an emphasis on being on the cutting edge).	(c)
5. La organización pone énfasis en la adquisición de nuevos recursos y la creación de nuevos retos. Se valoran el probar nuevas cosas y la búsqueda de nuevas oportunidades. (The organization emphasizes acquiring new resources and creating new challenges. Trying new things and prospecting for opportunities are valued).	(c)
6. La organización define el éxito en base a tener unos productos únicos y de última generación. Es líder en sus productos e innovadora. (The organization defines success on the basis of having the most unique or newest products. It is a product leader and innovator).	(e)
**Market items (Original OCAI)**	
1. La organización está muy orientada a resultados. Una de las mayores preocupaciones es completar el trabajo. La gente es muy competitiva y se orienta a conseguir los logros. (The organization is very results-oriented. A major concern is with getting the job done. People are very competitive and achievement-oriented).	Market-1
2. En general, se considera que el liderazgo de la organización constituye un enfoque racional, agresivo o orientado a resultados. (The leadership in the organization is generally considered to exemplify a no-non-sense, aggressive, results-oriented focus).	(c)
3. El estilo directivo de la organización se caracteriza por una fuerte competitividad, altas exigencias y los logros. (The management style in the organization is characterized by hard-driving competitiveness, high demands, and achievement).	Market-2
4. Lo que mantiene la organización unida es el énfasis en los logros y la consecución de los objetivos. (The glue that holds the organization together is the emphasis on achievement and goal accomplishment).	Market-3
5. La organización pone énfasis en las acciones competitivas y los logros. Alcanzar unos objetivos exigentes y ganar en el mercado son factores predominantes. (The organization emphasizes competitive actions and achievement. Hitting stretch targets and winning in the marketplace are dominant).	Market-4
6. La organización define el éxito en base a ganar en el mercado y estar por delante de la competencia. El liderazgo competitivo del mercado es primordial. (The organization defines success on the basis of winning in the marketplace and outpacing the competition. Competitive market leadership is key).	(c)
**Hierarchy items (Original OCAI)**	
1. La organización es un lugar muy controlado y estructurado. El trabajo de las personas suele estar regido por unos procesos formales. (The organization is a very controlled and structured place. Formal procedures generally govern what people do).	(c)
2. En general, se considera que el liderazgo de la organización constituye un ejemplo de coordinación, organización o eficiencia. (The leadership in the organization is generally considered to exemplify coordinating, organizing, or smooth-running efficiency).	(e)
3. El estilo directivo de la organización se caracteriza por la seguridad laboral, la conformidad, la predictibilidad y la estabilidad en las relaciones. (The management style in the organization is characterized by security of employment, conformity, predictability, and stability in relationships).	Hierarchy-1
4. Lo que mantiene la organización unida son las normas y políticas formales. Es importante que la organización mantenga un buen funcionamiento. (The glue that holds the organization together is formal rules and policies. Maintaining a smooth-running organization is important).	Hierarchy-2
5. La organización pone énfasis en la permanencia y la estabilidad. La eficiencia, el control y el buen funcionamiento de los procesos son importantes. (The organization emphasizes permanence and stability. Efficiency, control, and smooth operations are important).	Hierarchy-3
6. La organización define el éxito en base a la eficiencia. La fiabilidad de las entregas, una programación eficiente y los bajos costes de producción son fundamentales. (The organization defines success on the basis of efficiency. Dependable delivery, smooth scheduling, and *Low-Cost* production are critical).	Hierarchy-4

*(e) Discarded by the EFA due to complexity, (c) Discarded by the CFA.*

### Reliability

Reliability was good in both samples for the three factors. For n_1_ it was 0.86 for Clan, 0.82 for Market and 0.74 for Hierarchy. For n_2_ it was 0.87 for Clan, 0.74 for Market and 0.74 for Hierarchy.

Table 4 shows the values of each factor (Mean, SD) for the two samples and also the values of the items (Scale Mean if the item is deleted, Corrected item-total correlation, Cronbach’s alpha if the item is deleted). Table 5 shows the Cronbach’s alpha and Confidence intervals of each factor and the two samples.

**TABLE 4:** OCAI-12: values of the items (Mean, Standard deviation, Skewness, Kurtosis, Corrected item-total correlation, Cronbach’s alpha if the item is deleted) and values of each factor (Mean, Standard deviation) for the two samples.

	*n_1_* = 246						*n_2_* = 510					
Items CLAN Factor	(a)	(b)	(c)	(d)	(e)	(f)	(a)	(b)	(c)	(d)	(e)	(f)
Clan 1.	3.26	0.08	−0.36	−0.94	0.71	0.83	3.54	0.05	−0.57	−0.34	0.68	0.85
Clan 2.	3.51	0.08	−0.60	−0.57	0.65	0.85	3.57	0.05	−0.56	−0.32	0.70	0.84
Clan 3.	3.13	0.08	−0.30	−1.1	0.76	0.80	3.41	0.05	−0.40	−0.53	0.77	0.81
Clan 4.	3.08	0.08	−0.13	−1.1	0.72	0.82	3.48	0.05	−0.50	−0.62	0.74	0.83
**CLAN Factor**												
Mean			12.97					14.01				
SD			4.31					3.80				

**Items MARKET Factor**	**(a)**	**(b)**	**(c)**	**(d)**	**(e)**	**(f)**	**(a)**	**(b)**	**(c)**	**(d)**	**(e)**	**(f)**

Market 1.	3.47	0.08	−0.38	−0.75	0.59	0.80	3.97	0.04	−0.83	0.35	0.65	0.70
Market 2.	2.88	0.08	0.01	−1.1	0.69	0.75	3.66	0.05	−0.61	−0.09	0.58	0.74
Market 3.	3.36	0.07	−0.43	−0.56	0.62	0.78	3.81	0.04	−0.92	0.94	0.56	0.74
Market 4.	3.39	0.08	−0.45	−0.73	0.67	0.76	3.76	0.04	−0.85	0.55	0.57	0.74
**MARKET Factor**												
Mean			13.10					15.20				
SD			3.9					3.02				

**Items HIERARCHY Factor**	**(a)**	**(b)**	**(c)**	**(d)**	**(e)**	**(f)**	**(a)**	**(b)**	**(c)**	**(d)**	**(e)**	**(f)**

Hierarchy 1.	3.24	0.08	−0.31	−0.90	0.49	0.70	3.29	0.05	−0.42	−0.57	0.52	0.69
Hierarchy 2.	2.85	0.08	−0.03	−1.08	0.53	0.68	3.37	0.05	−0.39	−0.50	0.49	0.70
Hierarchy 3.	3.43	0.07	−0.57	−0.49	0.71	0.57	3.60	0.05	−0.70	0.10	0.61	0.64
Hierarchy 4.	3.43	0.08	−0.49	−0.67	0.41	0.74	3.68	0.05	−0.75	0.11	0.51	0.69
**HIERARCHY Factor**												
Mean			12.96					13.95				
SD			3.66					3.23				

*(a) Mean (M); (b) Standard deviation (SD); (c) Skewness (SK); (d) Kurtosis (KR); (e) Corrected item-total correlation; (f) Cronbach’s alpha if the item is deleted.*

**TABLE 5:** Cronbach’s alpha and Confidence intervals for each factor and the two samples.

	Clan		Market		Hierarchy	
	*n_1_* = 246	*n_2_* = 510	*n_1_* = 246	*n_2_* = 510	*n_1_* = 246	*n_2_* = 510
Cronbach’s alpha	0.86	0.87	0.82	0.74	0.74	0.74
Confidence intervals	0.83–0.89	0.85–0.89	0.78–0.85	0.70–0.78	0.68–0.79	0.70–0.78

### Evidence of Validity

Evidence of validity was calculated using Pearson correlations between the Clan, Market and Hierarchy scales with the contrast scales Performance measure, Prospector Strategy, Low-cost strategy, MORTN, MOPRO and the correlates Annual turnover and Number of employees. Table 6 shows 31 significant correlations for the two samples, of which two are negative. The highest positive correlations were MOPRO in F1 (Clan) (n_2_, *r* = 0.504, *p* < 0.01) and MORTN in F1 (Clan) (n_2_, *r* = 0.484, *p* < 0.01). Among those that least correlate positively were the two correlates, which are Annual turnover in F2 (Market) (n_2_, *r* = 0.147, *p* < 0.01) and Number of employees in F3 (Hierarchy) (n_2_, *r* = 0.134, *p* < 0.01). The two significant inverse correlations are Number of employees (n_1_, *r* = −0.161, *p* < 0.01) and Annual turnover (n_1_, *r* = −0.171, *p* < 0.05), both in F1 (Clan).

**TABLE 6:** OCAI-12: evidence of validity. Descriptive, reliability, confidence intervals, Pearson correlations between the three factors and the contrast scales and correlates, and Pearson correlations between the three factors.

		*n*_1_ = 246			*n*_2_ = 510		
		F1	F2	F3	F1	F2	F3
Mean		12.97	13.10	12.96	14.01	15.20	13.95
SD		4.31	3.9	3.66	3.80	3.02	3.23
Reliability		0.86	0.82	0.74	0.87	0.74	0.74
Confidence intervals		0.83–0.89	0.78–0.85	0.68–0.79	0.85–0.89	0.70–0.78	0.70–0.78
Scales	Performance measure (Babakus et al., 1996).	0.37**	0.24**	0.07	0.31**	0.39**	0.25**
	Prospector strategy (Slater and Olson, 2000).	0.29**	0.32**	0.04	0.34**	0.35**	0.22**
	Low-cost strategy (Slater and Olson, 2000).	−0.03	0.11	0.28**	0.06	0.17**	0.15**
	MORTN (Deshpandé and Farley, 1998).	0.46**	0.27**	0.20**	0.48**	0.43**	0.35**
	MOPRO (Narver et al., 2004).	0.45**	0.28**	0.08	0.50**	0.38**	0.31**
External Correlates	Number of employees	−0.16*	0.18**	0.09	−0.02	0.17**	0.13**
	Annual turnover	−0.17**	0.22**	0.07	−0.01	0.15**	0.08
F1		–	–	–	–	–	–
F2		0.06	–	–	0.46**	–	–
F3		0.12	0.16*	–	0.46**	0.48**	–

**p < 0.01 and *p < 0.05.

*F1, Clan; F2, Market; F3, Hierarchy (OCAI-12).*

## Discussion

### Summary and Discussion of the Results

The main aim of this study was to translate and adapt into Spanish the original 24-item OCAI scale (Cameron and Quinn, 2006) by analyzing its internal structure and evaluating reliability and validity. The OCAI is considered a good instrument for measuring organizational culture in English-speaking countries, but to date there has been no proper adaptation into Spanish.

Objective 1 was to analyze the internal structure of the translation into Spanish of the original 24-item OCAI. In our research the EFA reduced the internal structure from four to three factors. Afterward the CFA retained Clan, Market and Hierarchy (in each case reducing the number of items from six to four) and totally excluded *ad hoc*. The final model, named OCAI-12, has indicators with an acceptable fit (TLI = 0.93, CFI = 0.94, RMSEA = 0.07). Thus, this objective is only partially fulfilled and our study suggests that there are problems of construct equivalence between the English original OCAI and the Spanish translations. Effectively, in a previous attempt to translate the OCAI into Spanish conducted by Cerpa-Noya (2018) with a sample of 211 administrative workers in Metropolitan Lima and using EFA, the result obtained only two factors.

Objective 2 was to analyze the reliability of the scales. Reliability is considered correct when Cronbach’s alpha is 7 or higher. This objective is fulfilled, obtaining good reliabilities for the three factors and the two samples. Specifically, the reliability of the OCAI-12 for n_1_ was 0.86 (F1, Clan), 0.82 (F2, Market) and 0.74 (F3, Hierarchy) and for n_2_ it was 0.87 (F1, Clan), 0.74 (F2, Market) and 0.74 (F3, Hierarchy).

Objective 3 was to provide evidence of validity for the OCAI-12. Evidence of validity was calculated using Pearson correlations between the Clan, Market and Hierarchy scales with the contrast scales Performance measure, Prospector strategy, Low-cost strategy, MORTN, and MOPRO, and the correlates Annual turnover and Number of employees. Our results were largely in accordance with previous literature that supports the validity of the scales. We expected to find these five correlations:

(a) “The Hierarchy culture will have a smaller correlation with business performance than the Clan and Market cultures (Deshpandé et al., 1993; Cameron and Quinn, 2006; Jogaratnam, 2017).” This is fulfilled in our study. Effectively, in n_1_ we found no significant correlation between the Performance measure and the Hierarchy culture (F3), while the correlation with the Clan (F1) and Market (F2) cultures are *r* = 0.37 and *r* = 0.24 respectively, both with *p* < 0.01. In n_1_ we did find a significant correlation between the Performance measure and the Hierarchy culture (F3), with *r* = 0.25, but it is smaller than the correlation with the Clan (F1) and Market (F2) cultures, which are *r* = 0.31 and *r* = 0.39 respectively, the three with *p* < 0.01. According to the mentioned researchers, the Hierarchy culture is internally oriented and gets its stability from its systems and processes, but with the inconvenience of becoming a slow and rigid bureaucracy. These characteristics tend to hinder its competitiveness and lower its business performance.

(b) “The Hierarchy culture will have a positive correlation with the Low-cost strategy (Slater et al., 2010; Nase and Arkesteijn, 2018).” This is fulfilled in our study in n_1_ (*r* = 0.28, *p* < 0.01) and also in n_2_ (*r* = 0.15, *p* < 0.01). The explanation for this relationship is that the strong processes and systems that characterize the Hierarchy culture make it possible to lower the production costs, which supports a Low-cost strategy.

(c) “The Market culture will have a positive correlation with the Prospector strategy (Cameron and Freeman, 1991).” This is fulfilled in our study in n_1_ (*r* = 0.32, *p* < 0.01) and also in n_2_ (*r* = 0.35, *p* < 0.01). Cameron and Freeman (1991) argue that the Market culture is an externally oriented culture capable of understanding the clients’ needs and responding with new products and services.

(d) “The Hierarchy culture will have a smaller correlation with Market orientation than the Clan and Market cultures (Cameron and Quinn, 2006; Iglesias et al., 2011; Gao, 2017; Jogaratnam, 2017).” This is fulfilled in our study both for MORTN and MOPRO. In n_1_ the correlation between MORTN and the Hierarchy culture (F3) is *r* = 0.20, while the correlation with the Clan (F1) and Market (F2) cultures are higher, with *r* = 0.46 and *r* = 0.27 respectively. For the three cultures *p* < 0.01. In n_2_ the correlation between MORTN and the Hierarchy culture (F3) is *r* = 0.35, while the correlation with the Clan (F1) and Market (F2) cultures are also higher, with *r* = 0.48 and *r* = 0.43 respectively. For the three cultures *p* < 0.01. In turn, in n_1_ the correlation between MOPRO and the Hierarchy culture (F3) is not significant, while the correlations with the Clan (F1) and Market (F2) cultures are *r* = 0.45 and *r* = 0.28 respectively. For Clan and Market *p* < 0.01. In n_2_ the correlation between MOPRO and the Hierarchy culture (F3) is *r* = 0.31, while the correlations with the Clan (F1) and the Market (F2) cultures are higher, with *r* = 0.50 and *r* = 0.38 respectively. For the three cultures *p* < 0.01. According to the mentioned researchers, the Hierarchy culture is internally oriented and competes with a strong focus on its systems and processes, but with the inconvenience of lowering its customer orientation.

(e) “The Clan culture will have a negative correlation with the size of the company (Cameron and Quinn, 2006).” This is partially fulfilled in our study, where in n_1_ the correlation of the Clan culture (F1) is negative both for the Number of employees (*r* = −0.16, *p* < 0.05) and the Annual turnover (*r* = −0.16, *p* < 0.01). However, in n_2_ the correlation is not significant. Cameron and Quinn (2006) argue that the Clan culture is stronger during the first stages of the creation of companies, where the small number of people permits very close relationships to be established. As companies grow and increase their revenues and the number of employees the Clan culture tends to decrease due to the difficulty of maintaining the family atmosphere and strong ties. Nevertheless the “relational theory of society” built with diverse contributions and mentioned by Pellicano et al. (2016) asserts that human reality is relational by essence and gives enormous importance to the social relationship. This social relationship could be more robust in the Peruvian sample and could explain why Clan culture do not decrease with the size of its companies.

In summary, the translation and adaptation into Spanish of the OCAI (Cameron and Quinn, 2006) has given rise to the OCAI-12, with a three-factor structure that retains the Clan, Market and Hierarchy factors, but fully excludes the *ad hoc* factor. The Confirmatory Factor Analysis shows acceptable indicators and the reliabilities and indications of validity are also good. This study represents the largest activity to date for adapting the OCAI (Cameron and Quinn, 2006) into Spanish using EFA and CFA. It also shows the difficulty in adapting this scale from English into Spanish. Despite the rigor of the translation method, the different meanings of the words and concepts make it difficult to construct equivalence and it was necessary to reduce the original OCAI to the point of completely discarding the *ad hoc* factor. Previous studies have given support to the internal four-factor structure with the 24 items in English and found proper reliability and validity (Quinn and Spreitzer, 1991; Kalliath et al., 1999; Heritage et al., 2014). Adaptations to Italian (Di Stefano and Scrima, 2016) and Iranian (Abbasi et al., 2013) have also found the four-factor structure. The reason why the *ad hoc* culture cannot be properly translated into Spanish is unclear, but a possible explanation to study could be related to Uncertainty avoidance (Hofstede, 1984). This is the extent to which a society feels threatened and anxious when they confront ambiguous and unknown situations and thus the point to which they try to escape from them. Uncertainty avoidance is much higher in Spain and most Latin American countries than in the United States, the United Kingdom and Australia. There are items in the OCAI where taking risks is associated with the idea of being innovative and entrepreneurial (e.g., People are willing to stick their necks out and take risks). As risk taking is not so desirable in Spain and Latin America, the *ad hoc* scale in Spanish may fail. The meaning of other complex concepts in the *ad hoc* scale (e.g., entrepreneur, freedom, being on the cutting edge, etc.) could reduce the covariance of the six items even more.

## Conclusion

This study describes the translation and adaptation of the Organizational Culture Assessment Instrument (Cameron and Quinn, 2006) into Spanish, giving rise to the reduced scale named OCAI-12. The CFA shows indicators with an acceptable fit and it also has good reliability indexes. Moreover, the scale provides evidence of validity given that it has correlations with five different scales and two correlates. All of this indicates that the OCAI-12 scale is a suitable instrument in Spanish for evaluating organizational culture.

Our study findings drive us to propose two implications to theory. We indicate that the Clan, Market and Hierarchy are types of organizational culture that have a correct construct equivalence when translated and adapted from English into Spanish. Nevertheless, the *ad hoc* culture have important problems of construct equivalence, suggesting enormous differences in the meaning and connotations of the concepts included in the measurement scale.

Our results also have a practical implication for academics and practitioners alike, who can now use the OCAI-12 measurement instrument, but are also advised to be careful with *ad hoc* translations into Spanish.

The results of our work should be considered in light of several limitations. We would also like to make some suggestions for future research. Firstly, our data were obtained with non-probabilistic sampling of Spanish and Peruvian managers. We recommend extending the research to other employee profiles. Secondly, the OCAI-12 has reduced the Clan, *ad hoc* and Market scales from six items to four. A new study could be conducted to increase the number of items and make the instrument more robust (Lloret-Segura et al., 2014). Thirdly, a scale in Spanish could be constructed for the *ad hoc* culture because with EFA and CFA the six items have been excluded from the model. Measuring this culture, which is characterized by its innovative capacity, is especially important for business competitiveness. Finally, complementary studies should extend the sample to each Latin American country to ensure that the conclusions can be extrapolated to a national level.

## Data Availability Statement

The raw data supporting the conclusions of this article will be made available by the authors, without undue reservation.

## Ethics Statement

Ethical review and approval was not required for the study on human participants in accordance with the local legislation and institutional requirements. The patients/participants provided their written informed consent to participate in this study.

## Author Contributions

JA-S, MB-C, and JB-G: conceptualization. JA-S, M-JS-F, EV, and JB-G: methodology. JA-S, MB-C, M-JS-F, and JB-G: writing—original draft preparation. EV: manuscript revision. All authors have read and agreed to the published version of the manuscript.

## Conflict of Interest

The authors declare that the research was conducted in the absence of any commercial or financial relationships that could be construed as a potential conflict of interest.

## Publisher’s Note

All claims expressed in this article are solely those of the authors and do not necessarily represent those of their affiliated organizations, or those of the publisher, the editors and the reviewers. Any product that may be evaluated in this article, or claim that may be made by its manufacturer, is not guaranteed or endorsed by the publisher.
